# Lésions tomodensitométriques chez les patients hospitalisés pour pneumonie à COVID-19 lors de la première vague de la pandémie à SARS-CoV-2 aux Cliniques Universitaires de Kinshasa (RDC)

**DOI:** 10.11604/pamj.2021.39.230.29311

**Published:** 2021-08-10

**Authors:** Frederick Tshibasu Tshienda, Michel Lelo Tshikwela, Jean-Robert Makulo Risasi, Hippolyte Nani Tuma Situakibanza, Randa Salem, Patrick Sekele Marob Ndjock, René Ngiyulu Makwala, Antoine Molua Aundu, Joseph Bodi Mabiala, Jean-Marie Kayembe Ntumba, Benjamin Longo-Mbenza

**Affiliations:** 1 Service d´Imagerie Médicale, Cliniques Universitaires de Kinshasa, Kinshasa, République Démocratique du Congo,; 2 Service de Néphrologie, Cliniques Universitaires de Kinshasa, Kinshasa, République Démocratique du Congo,; 3 Service des Maladies Infectieuses, Cliniques Universitaires de Kinshasa, Kinshasa, République Démocratique du Congo,; 4 Service d´Imagerie Médicale, Hôpital Universitaire Fatouma Bourkiba, Monastir, Tunisie,; 5 Département d´Odontostomatologie, Cliniques Universitaires de Kinshasa, Kinshasa, République Démocratique du Congo,; 6 Département de Pédiatrie, Cliniques Universitaires de Kinshasa, Kinshasa, République Démocratique du Congo,; 7 Service de Pneumologie, Cliniques Universitaires de Kinshasa, Kinshasa, République Démocratique du Congo,; 8 Service de Cardiologie, Cliniques Universitaires de Kinshasa, Kinshasa, République Démocratique du Congo

**Keywords:** COVID-19, prise en charge, tomodensitométrique, Kinshasa, COVID-19, management, CT, Kinshasa

## Abstract

**Introduction:**

l´objectif principal de cette étude, consiste à ressortir les aspects tomodensitométriques thoraciques (TDM) observés lors de la première vague de la pandémie à SARS-CoV-2 chez les patients hospitalisés pour pneumonie à COVID-19 aux Cliniques Universitaires de Kinshasa (CUK).

**Méthodes:**

étude descriptive portant sur les examens TDM thoraciques des 26 patients hospitalisés pour pneumonie à COVID-19 aux CUK sur une période de 9 mois, allant de 17 mars au 17 novembre 2020. Un appareil TDM 16 barrettes de marque Hitachi a été utilisé chez tous nos patients. Après analyse, les lésions trouvées ont été réparties en lésions évocatrices et non évocatrices de l´infection à SARS-CoV-2.

**Résultats:**

l´âge médian des patients était de 53,02 ans. Le sexe masculin était le plus touché avec 76,9%. La détresse respiratoire était le symptôme clinique le plus rencontré avec 61,5%. Comme comorbidité, l´hypertension artérielle (HTA) et l´insuffisance rénale étaient les plus retrouvées avec 30% et 6%. Les opacités en Verre dépoli bilaterales avec prédominance périphérique étaient prédominantes (69,2%), suivies des condensations (57,7%) et des crazy paving (19,2%). Le stade sévère était le plus fréquemment rencontré (34,61%). L´embolie pulmonaire distale et proximale était la complication la plus fréquente (11,5%). Parmi les pathologies associées la pleurésie et l´HTA pulmonaire étaient les plus rencontrées (30%, 8%). La majorité de nos patients, ont présentés des lésions parenchymateuses pulmonaires correspondant au stade TDM précoce de la maladie (50%).

**Conclusion:**

les lésions TDM évocatrices de COVID-19, observées aux CUK lors de la première vague sont dominées par les plages d´opacités en verre dépoli, suivies des condensations parenchymateuses non systématisées et des crazy paving. Les lésions atypiques moins observées, étaient constituées des condensations pseudo-nodulaires, d´atteinte unilatérale, d´atteinte péribronchovasculaire et d´infection sur le poumon remanié. Le stade TDM sévère était la plus fréquente. L´embolie pulmonaire proximale et distale était la complication la plus rencontrée. Les aspects trouvés corroborent majoritairement les observations de la littérature.

## Abstract (en)

**Introduction:**

the main purpose of this study is to describe chest computed tomography (CT) findings in 26 patients hospitalized with COVID-19 pneumonia during the first wave of the SARS-CoV-2 pandemic at the University Clinics in Kinshasa (UCK).

**Methods:**

we conducted a descriptive study of chest CT findings in 26 patients hospitalized with coronavirus pneumonia at the UCK over a 9-month period, from March 17 to November 17, 2020. Hitachi - CT-scanner 16 slice was used in all our patients. After analyzing lesions, these were divided into lesions suggestive and non-suggestive of SARS-CoV-2 infection.

**Results:**

the average age of patients was 53.02 years. Male sex was the most affected (76.9%). Respiratory distress was the most common clinical symptom (61.5%). Arterial hypertension and renal failure were the most common comorbidities (3O% and 6%). Bilateral ground-glass opacities, with a predominantly peripheral distribution, accounted for 69.2% of cases, followed by condensations (57.7%) and crazy paving (19.2%). Severe COVID-19 was most frequently found (34.61%). Distal and proximal pulmonary embolism was the most common complication (11.5%). Among the associated diseases, pleurisy and pulmonary PAH were most frequently found (30.8%). The majority of our patients had parenchymal lung lesions, corresponding to early-stage disease on CT (50%).

**Conclusion:**

at the UCK, during the first wave of SARS-CoV-2 pandemic, lesions on CT suggestive of COVID-19 were dominated by plaque-like ground-glass opacities, followed by nonsystematized parenchymatous condensations and crazy paving. The less observed atypical lesions consisted of unilateral, peribronchovascular pseudo-nodular condensations and infection in the remodeled lung. Severe COVID-19 was the most common CT finding. Proximal and distal pulmonary embolism was the most common complication. This study highlights that these findings are consistent with those reported in the literature.

## Introduction

Mois de décembre de l´an 2019, une nouvelle maladie responsable d´une série des cas de pneumonie virale provoquée par un nouveau coronavirus est apparue en Chine dans la ville de Wuhan (province de Hubei) [1,2]. Cette dernière s'est rapidement propagée sur tous les continents. Cette maladie a conduit l'Organisation mondiale de la santé (OMS) à la déclarée urgence de santé publique de portée internationale le 30 janvier 2020 puis pandémie le 11 mars 2020 [1,3]. Ce coronavirus, identifié sur les prélèvements des secrétions des voies respiratoires supérieurs, a été nommé SARS-CoV-2 signifiant Severe Acute Respiratory Syndrom CoronaVirus-2 par international Committee On Taxonomy of Viruses (l'ICTV) [1]. La maladie engendrée par ce virus a été dénommée COVID-19 désignant Corona Virus Disease 2019 par l'OMS. Le SARS-CoV-2 appartient à la famille des Coronaviridae, tout comme le SARS-CoV et le MERS-CoV qui furent responsables d'épidémies de grande ampleur en 2003 et 2012 au Moyen Orient [1].

En République Démocratique du Congo (RDC) le premier cas de COVID-19 a été déclaré le 07 mars 2020. Au moment de la rédaction de ce papier (23/Novembre/2020); le pays a enregistré depuis le début de l´épidémie, 12179 cas confirmés, un cas probable, dont trois cents vingt-huit décès, soit une létalité globale de 2,7%. Les professionnels de santé représentaient 15,3% des cas pour lesquels la profession a été renseignée. Leurs âges, pour les trois cents un professionnels dont l´information était connue, variaient de 22 à 88 ans avec une médiane de 41,5 ans [4]. La présentation clinique la plus typique du COVID-19 est celle d'une infection respiratoire fébrile avec toux sèche, dyspnée, fatigue et myalgies. Environ 8O% des cas sont bénins, 15-20% graves et 2-3% mortels [5-7]. Vu l´indisponibilité de thérapeutique spécifique jusqu´à ce jour, le diagnostic précoce de cette pathologie s´impose afin d'isoler les sujets infectés et limiter sa propagation. La modalité diagnostique référentielle reste la recherche d'ARN viral par RT-PCR (reverse transcriptase-polymérase chain reaction) à partir d'écouvillonnages naso-pharyngés. Outre, sa spécificité qui est excellente, sa sensibilité reste imparfaite 60 à 70%, car dépendante de la qualité du prélèvement et du taux de réplication virale au sein des voies respiratoires supérieures, occasionnant des faux négatifs autour de 40 à 30% [8,9].

Le scanner thoracique s'est rapidement imposé depuis le début de l´épidémie comme un outil diagnostic intéressant, compte tenu de la présentation assez caractéristique des lésions de COVID- 19 [2,10]. Des séries chinoises et italiennes avaient rapporté une sensibilité de la TDM au début de la pandémie autour de 25% et 56% [9,11]. La sensibilité du scanner pour le diagnostic de COVID-19 est supérieure à 90 % à ce jour, les faux négatifs, scanners normaux alors que la maladie est présente; correspond généralement à des patients présentant des symptômes depuis moins de 3 jours [9,12]. La spécificité du scanner est plus variable. L´objectif principal de cette étude, consiste à ressortir les lésions TDM vues lors de la première vague de la pandémie à SARS-CoV-2 chez les patients hospitalisés pour pneumonie à COVID-19 aux CUK.

## Méthodes

**Cadre de l'étude**: la présente étude a été réalisée au centre de prise en charge de COVID-19 situé des Cliniques Universitaires de Kinshasa, situées sur le Mont-Amba, dans la Commune de Lemba. Les CUK occupent une superficie de 27110 m^2^ et deux voies d'accès y sont possibles entre autre: l'avenue de l'Université et l'avenue de la Foire au niveau du Rond-Point-Ngaba.

**Type d´étude**: il s'agit d'une étude monocentrique, transversale descriptive, réalisée sur une période de neuf mois, soit du 17 mars au 17 novembre 2020.

**Participants à l'étude et conception de l'étude**: étaient inclus, tout patient de nationalité congolaise, homme ou femme, tout âge confondu, hospitalisé pour infection à SARS-CoV-2 lors de la première vague aux CUK avec une RT-PCR positive et une TDM thoracique avec protocole low dose. Par contre, tout patient hospitalisé pour infection à SARS-CoV-2 aux CUK lors de la première vague sans résultat de la RT-PCR, sans TDM thoracique avec protocole low dose ou ayant réalisé uniquement les radiographies thoraciques ont été exclus de la présente étude. Tous les patients ont été pris en charge conformément au protocole national de prise en charge édicté par le Secrétariat Technique National de la Riposte contre la COVID-19 en RDC. Un appareil TDM de marque HITACHI 16 barrettes de modèle Eclos, mis en service en 2011 a été utilisé chez tous nos patients. Comme paramètres d´acquisition; nous avons utilisé: 120 kV, 100 à 150 mA, 0,6 mm de collimation et 1:1 de pitch. Le champ d´exploration thoracique couvrait l'apex pulmonaire au diaphragme sur le plan axial pris en respiration libre avec les patients en décubitus dorsal. Les images TDM natives ont été obtenues avec des coupes de 2,5 mm puis reconstruites avec une collimation de 1,25 mm avec un algorithme standard, puis envoyées à l'archivage des images et système de communication (PACS) pour l'analyse. Les images TDM ont été évaluées en utilisant une fenêtre pulmonaire avec un niveau de fenêtrage de -600 HU et une largeur de 1500 HU. Le niveau de fenêtrage des tissus mous était de 40 HU et une largeur de 300 HU. Au total six examens avaient bénéficié de l´injection du produit de contraste iodé à une dose de 1 à 1,5 ml/kg de poids corporel après obtention du bilan de la fonction rénale (urée et créatinine) et réhydratation du patient. Toutes les images ont été stockées dans le PACS et examinées par trois médecins radiologues thoraciques expérimentés. Les différents patterns radiologiques thoraciques ci-dessous, évocateurs du diagnostic de COVID-19 ont été passés en revue entre autres: (a) les opacités en verre dépoli: zone de surdensité parenchymateuse pulmonaire n´effaçant pas les vaisseaux pulmonaires, (b) le crazy paving: apparition des réticulations intra et interlobulaires aux seins des verres dépoli, (c) les condensations parenchymateuses: zone de surdensité parenchymateuse pulmonaire systématisée ou non, effaçant les vaisseaux pulmonaires, (d) les lésions atypiques de COVID-19: lésions autres que des opacités en verre dépoli sous pleurales, des Crazy paving ainsi que des condensations systématisées, (e) la gravité lésionnelle était définie par l´étendue des lésions; cette dernière permet de grader l´atteinte en: COVID-19 minime: étendue lésionnelle < 10%, COVID-19 moderée: étendue lésionnelle comprise entre 10-25%; COVID-19 étendue : étendue lésionnelle comprise entre 25-50%, COVID-19 sévère: étendue lésionnelle comprise entre 50-75% et COVID-19 critique: étendue lésionnelle >75%; (f) l´évolution de l´atteinte pulmonaire était stadifiée en: stade précoce: jusqu´aux 4 premiers jours, stade intermédiaire: 5 à 8 jours, stade tardif: 8 à 13 jours, stade très tardif: au-delà de 14 jours, (g) les complications pulmonaires étaient définies par l´apparition de l´embolie pulmonaire et ou du syndrome de détresse respiratoire aigüe enfin, (h) les pathologies associées: la présence des pathologies autres que les complications ci- haut citées.

**Analyse des données**: un ordinateur de marque Dell muni des logiciels : Epidata 3.1, SPSS (Statistical package for Social Sciences) version 18, Excel et Word 2010 a servi pour la saisie et les analyses statistiques. Les variables quantitatives ont été exprimées sous forme de moyennes, écart-types ou en médiane avec les extrêmes et les variables qualitatives sous forme de pourcentage. Les résultats sont présentés sous- formes de tableaux et figures. Le principe de confidentialité était rigoureusement observé lors de la collecte, de la saisie et de l´analyse des données en utilisant l´anonymat.

## Résultats

**Figure 1:**
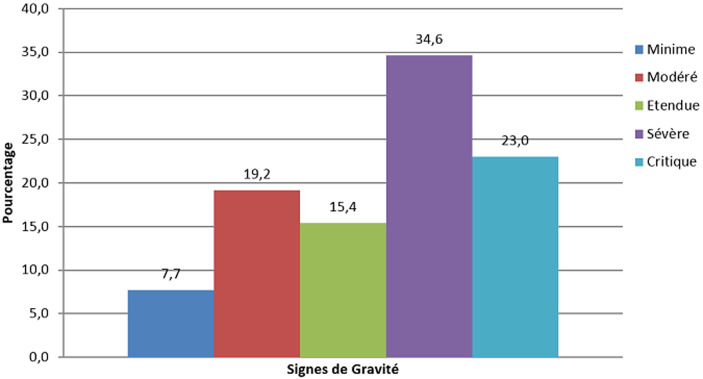
répartition des lésions de COVID-19 en fonction de l´étendue sur le parenchyme pulmonaire; COVID-19 minime < 10%, COVID-19 modérée 10-25%, COVID-19 étendue 25-50%, COVID-19 sévère 50-75%, COVID-19 critique >75%

**Figure 2:**
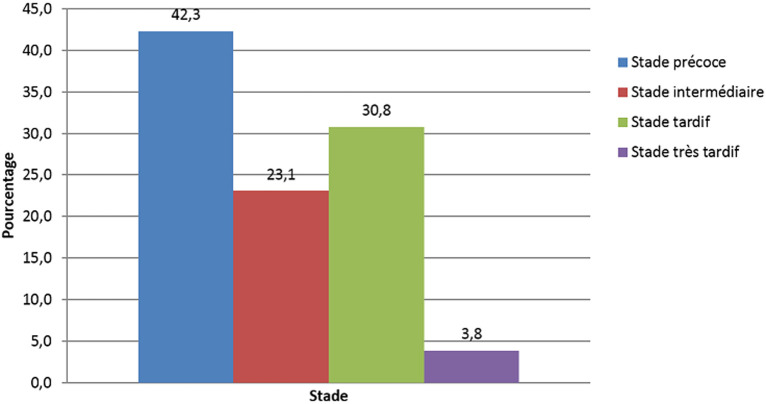
répartition des lésions de COVID-19 en fonction des stades évolutifs sur la TDM thoracique: a) stade précoce: jusqu´aux 4 premiers jours, opacités en verre dépoli, b) stade intermédiaire (5-8j): extension des plages de verre dépoli, confluence, organisation de condensations et apparition d´opacités linéaires, c) stade tardif (8-13j): diminution des plages en verre dépoli au profit des condensations et des opacités linéaires, d) stade tardif (au-delà de 14j): régression progressive des anomalies

L´âge médian des patients était de 53,02 ans avec les âges extrêmes compris entre 4 mois 8 jours pour le moins âgé et 79 ans pour le plus âgé. Le sexe masculin était le plus touché avec 76,9% pour un sex-ratio de 0,3 (Tableau 1). Sur le plan clinique; la détresse respiratoire était la manifestation clinique la plus rencontrée 61,5%, et comme comorbidités l´hypertension artérielle et l´insuffisance rénale venaient en tête avec 30,6% (Tableau 1). Sur un effectif de deux cents dix patients, vingt-six patients soit 12,38 % avaient réalisés un examen TDM. Tous les vingt-six scanners thoraciques étaient pathologiques 72,22% (Tableau 2). La DLP (dose length product) moyenne était de 11O mGy.cm avec des extrêmes de 79 et 14O mGy.cm. En rapport avec les lésions évocatrices de COVID-19, les opacités en verre dépoli étaient prédominantes, 61,53%. Ces opacités étaient: multifocales à 65,4%, bilatérales à 69,2%, asymétriques à 65,4%, périphériques à 69,2%, basales à 38,5 %, centrales à 23,1% et mixtes 7,7% (Tableau 2). Elles étaient suivies des condensantes parenchymateuses pulmonaires 57,7%; les condensations en plages étaient prédominantes; suivies de celles en bandes puis nodulaires (Tableau 2). Quant à leurs localisations; l´atteinte des lobes pulmonaires inferieurs était prédominante chez 69,23%, suivie des lobes supérieurs. Les atteintes lobaires moyennes et pansegmentaires (tous les segments) étaient les moins rencontrées dans cette série. L´aspect en crazy paving: traduisant l´apparition des fines réticulations au sein des plages de verre dépoli était retrouvé chez 19,2% des cas (Tableau 2). Les lésions parenchymateuses atypiques étaient présentes chez 38,46% et parmi ces lésions; les condensations pseudo nodulaires étaient présentes chez 11,5%, l´atteinte parenchymateuse unilatérale présente chez 11,5%, l´atteinte péri bronchovasculaire présente chez 11,5% et l´infection sur poumon remanié (exemple: fibrose, emphysème) présente chez 3,8% (Tableau 2). L´évaluation de l´étendue lésionnelle avait révélé une prédominance de l´atteinte sévère chez 34,61%, suivie de l´atteinte critique chez 23,007 %, modérée chez 19,23%, étendue chez 15,36%, enfin minime chez 7,69% des cas (Figure 1). L´embolie pulmonaire a été la complication la plus rencontrée dans cette série avec 11,5 %. Elle était proximale chez 3,8% des cas et distale chez 7,7% des cas. Le syndrome de détresse respiratoire aigu (SDRA) était présent chez 7,7% (Tableau 3). Concernant les pathologies associées, nous avons noté une prédominance d´épanchement pleural liquidien et d´hypertension arterielle pulmonaire chez 30,8% (Tableau 3). L´excavation parenchymateuse apicale droite sur tuberculose post-primaire était trouvée chez 3,8%, la miliaire tuberculeuse chez 3,8% ainsi que les bronchiectasies trouvées chez 3,8% (Tableau 3). L´évolution des lésions parenchymateuses pulmonaires était basée sur les stades évolutifs des lésions à la TDM. Il ressort de ce qui précède, que la majorité de nos patients étaient examiné aux stades TDM précoce de la maladie: 50%, suivi du stade intermédiaire: 30,76%, du stade tardif: 15,38% et enfin du stade très tardif: 3,84% (Figure 2).

**Tableau 1:** analyse univariée des tranches d´âge, de sexes, des antécédents ainsi que de renseignements cliniques des patients

Variables	Effectifs (n=26)	Pourcentage (%)
**Intervalle d´âge (ans)**		
> 60	12	46,2
40 â 59	7	26,9
20 à 39	5	19,2
< 19	2	7,7
**Sexe**		
Féminin	6	23,1
Masculin	20	76,9
**Symptômes**		
Détresse respiratoire	16	61,5
Fièvre	2	7,7
Tuméfaction de la cuisse	1	3,8
Pollakiurie	1	3,8
Non rapporté	6	23,1
**Antécédents(Comorbidités)**		
Atteinte rénale	3	11,5
HTA + Atteinte rénale	3	11,5
HTA+DS+Atteinte rénale	1	3,8
HTA	1	3,8
DS + Amputation	1	3,8
Adénocarcinome de la prostate	1	3,8
Grossesse + TBC	1	3,8
Grossesse	1	3,8
Non rapporté	14	53,8

**Tableau 2:** analyse univariée des lésions TDM typiques et atypiques de COVID-19 trouvées à la TDM thoracique

Variables	Effectifs (n=26)	Pourcentage (%)
**Lésions typiques Opacités en verre dépoli**		
Multifocales	17	65,4
Bilatérales	18	69,2
Asymétriques	17	65,4
Prédominance périphérique	18	69,2
Prédominance centrale	6	23,1
Mixtes	2	7,7
Postérieures	16	61,5
Basales	10	38,5
**Crazy Paving**		
Présent	5	19,2
Condensations		
Présente	15	57,7
**Lésions atypiques**		
Condensations pseudo nodulaires		
Présente	3	11,5
**Atteintes unilatérales**		
Présente	3	11,5
**Atteintes péri broncho-vasculaires**		
Présente	3	11,5
**Infection sur poumon remanié**		
Présente	1	3,8

**Tableau 3:** analyse univariée des pathologies associées et des complications pulmonaires de COVID-19

Variables	Effectifs (n=26)	Pourcentage (%)
**Pathologies associées**		
Hémothorax	1	3,8
Excavation	1	3,8
Miliaire bi apicale	1	3,8
Bronchiectasie	1	3,8
Ascite	1	3,8
Polykystose hépatorénale	1	3,8
Dissection artère fémorale + Hématome de la cuisse	1	3,8
Non rapporté	11	42,3
**Complications**		
**Embolie Pulmonaire**		
EP Proximale et Distale	3	11,5
EP Distale	2	7,7
Pleurésie	4	15,4
Non rapporté	20	76,9
**SDRA**		
Présente	2	7,7
Absente	24	92,3

## Discussion

**Figure 3:**
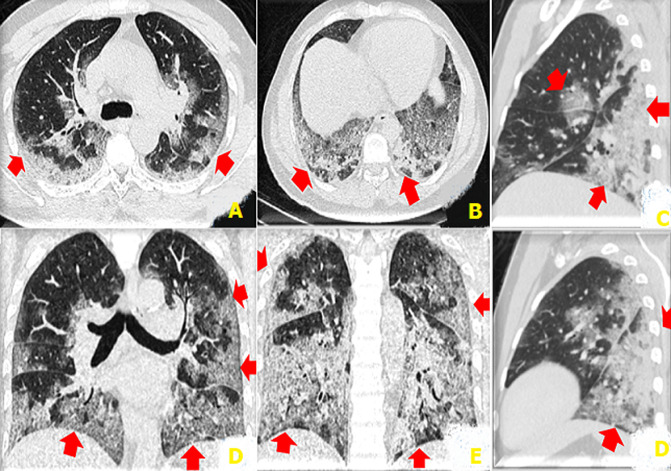
TDM thoracique en filtre parenchymateux: A et B: coupes axiales, C et D: reconstructions coronales, E et F: reconstructions sagittales: opacités en crazy paving associées à des condensations postéro basales, épargnant les segments apicaux et apico-dorsal

**Figure 4:**
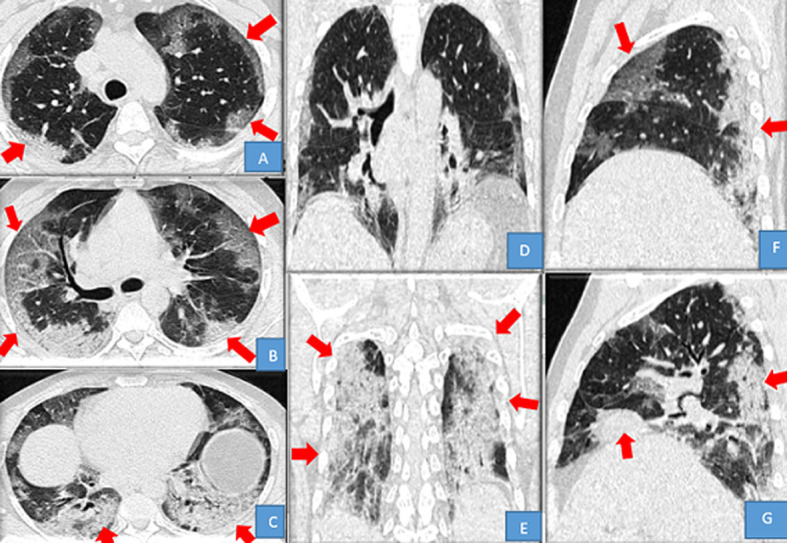
TDM thoracique en filtre parenchymateux: A, B et C: coupes axiales, D et E: reconstructions coronales, F et G: reconstructions sagittales: opacités en crazy paving associées à des condensations postéro-basales

L'âge médian des patients avec des lésions évocatrices de COVID-19 était de 53,02 ans avec les âges extrêmes compris entre 4 mois 8 jours et 79 ans. Néanmoins la tranche d'âge de 50 ans ou plus était celle avec les plus des lésions évocatrices de COVID-19; ce qui corrobore les données de la littérature chez les patients atteints de COVID-19 [1]. La moyenne des DLP dans cette série était de 14O mGy.cm; 2 fois moins élevée que les niveaux de références diagnostiques européennes pour une TDM thoracique (348 mGy.cm) [9]. L´hors de la première vague; les scanners thoraciques ont été moins réalisés 12,38%, au profit des radiographies standards; ce faible taux de réalisation des TDM thoraciques peut être justifié non seulement par la politique de prise en charge du départ mise en place par l´équipe de riposte; mais également par manque d´accessibilité au scanner pour la majorité des patients atteints de COVID-19 en milieux hospitalier de Kinshasa. Se référant aux lésions évocatrices de COVID-19 vues au scanner dans notre série; les opacités en verre dépoli étaient présentes chez 61,53%; elles étaient multifocales chez 87,5%, bilatérales chez 87,5%, asymétriques chez 87,5%, périphériques chez 75%; basales chez 56,25%, enfin centrales et mixtes à égalité chez 12,5% pour chacune de localisation. Ces opacités en verre dépoli peuvent se retrouvées dans d´autres pathologies; mais apparaissent à partir du 4^e^ jour dans l´infection à SARS-CoV-2, puis évoluent vers un aspect en crazy paving à partir du 8^e^ jour (Figure 3). Elles constituent le premier pattern lésionnel; lorsqu´elles sont périphériques, basales, bilaterales, asymétriques et multifocales. Les études de Niang *et al*. ainsi que de Guan *et al*. [13,14] ont sur 57 patients retrouvaient une même proportion de lésions de COVID-19 à la TDM avec 88,7% et les opacités en verre dépoli ont été trouvées chez tous leurs patients. Ceci démontre la sensibilité du verre dépoli et surtout de sa distribution sous-pleurale pour le diagnostic de COVID-19 malgré le fait qu'il ne soit spécifique d'aucune pathologie. Dans le même ordre d´idée; les condensations parenchymateuses pulmonaires ont été retrouvées en seconde position chez 57,7 % des patients parmi lesquelles; les condensations en plages venaient en tête avec 76,92%, suivies de celles en bandes (46%) ainsi que nodulaires (23,07%) (Figure 4). Elles étaient prédominantes aux lobes pulmonaires inferieurs chez 18 patients soit 69,23%, suivie des lobes supérieurs. Les atteintes lobaires moyennes et pansegmentaires (tous les segments) étaient les moins rencontrées dans cette série. Nos données corroborent les données de la littérature; notamment l´étude de Lodé sur l´imagerie de la pneumonie à COVID-19 [10].

Cette prédominance lobaire inferieure, peut s´expliquer par le fait que ces zones sont les plus perfusées et les moins ventilées au profit des lobes supérieurs. L´aspect en crazy paving; traduisant la superposition de verre dépoli et de réticulations intra-lobulaires et/ou de condensations parenchymateuses plus ou moins rétractiles ou encore l´apparition des fines réticulations au sein des plages de verre dépoli [10-12,15] était retrouvé chez 5 patients; soit 19,23% des cas. Le faible pourcentage de ces deux lésions chez nos patients s'explique par le fait que ces lésions apparaissent plus tardivement dans l'évolution de la COVID-19 entre le 8^e^ et le 9^e^ jour; ou en cas de surinfection pour ce qui est de la condensation pulmonaire [12,15]. La majorité de nos patients étaient essentiellement diagnostiqués aux stades précoce et intermédiaire de la maladie (avant le 8^e^ jour) c´est-à-dire avant l´apparition des crazy paving. Les lésions parenchymateuses atypiques du COVID-19 rapporté dans près de 10% des cas dans la littérature [10]; étaient présentes chez 10 de nos patients soit 38,46% et parmi ces lésions; nous avons noté: cinq cas des condensations pseudo nodulaires soit 19,23%, trois cas d´atteinte parenchymateuse unilatérale soit 11,53% et deux cas d´atteinte péribronchovasculaire soit 7,69%. Signalons que les présentations unilatérales sont possibles dans 20 à 30% des cas environ, ces dernières sont généralement rencontrées à un stade précoce avant que les lésions ne se bilatéralisent [9,10,16,17]. Les atteintes à prédominance péribronchovasculaire ou parfois apicale ont été également rapportées par les données de la littérature [10,18]. L´évaluation de l´étendue lésionnelle dans cette série avait révélé une prédominance des atteintes sévère et critique respectivement à 34,61% et 23,07% contrairement aux autres atteintes lésionnelles: modérée à 19,23%, étendue à 15,36%, enfin minime chez à 7,69% des cas. Elle reste le principal signe tomodensitométrique de gravité des anomalies parenchymateuses sur le scanner initial. Nombreuses études rapportent une corrélation entre l'extension des lésions et la sévérité clinique [10,19-21].

**Figure 5:**
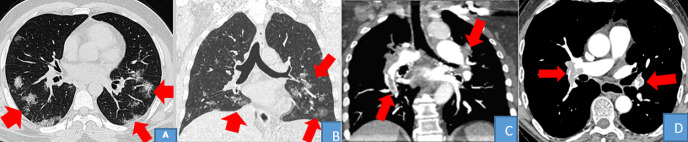
TDM thoracique en filtre parenchymateux: A (coupe axiale); B (coupe coronale) et en filtre médiastinal C (coupe axiale)-D (coupe coronale) après injection du produit de contraste iodé: opacités en Grounds class+ embolie pulmonaire proximale bilaterale plus marquée à droite

La Société d'imagerie thoracique (SIT) recommande la gradation de l'atteinte parenchymateuse sur base d´une classification visuelle en 5 stades, basée sur le rapport entre le parenchyme pulmonaire sain sur le parenchyme lésé; cette dernière repartie en: atteinte absente ou minime < 10%, modérée: 10-25%, étendue: 25-50%, sévère: 50-75% et critique >75% [10,22]. À côté de l'extension lésionnelle, la densité du parenchyme est également un marqueur de gravité, les condensations parenchymateuses apparaissant plus étendues que le verre dépoli chez les patients les plus graves [20]. L´embolie pulmonaire a été la complication la plus rencontrée dans cette série soit 26,92% (Figure 5). La localisation thrombotique distale étaient prédominante chez quatre patients soit 15,38% des cas. Un seul cas de syndrome de détresse respiratoire aigu a été retrouvé dans cette série. Le faible taux des patients ayant présenté une embolie pulmonaire peut être justifiée par le fait que la majorité des scanners thoraciques étaient réalisés sans produit de contraste. En outre cette localisation distale pouvait s´expliquer par le fait que; cette dernière s´accompagne d´une forte morbidité, tandis que la localisation proximale est pourvoyeur de la forte mortalité. Comme autres complications, nous avons relevées la prédominance d´épanchement pleural liquidien, talonné par l´hypertension artérielle pulmonaire et l´épanchement péricardique. Le nodule pulmonaire angiocentré, l´excavation apicale droite sur tuberculose post-primaire, la miliaire tuberculeuse, les bronchiectasies ainsi que l´hématome de la cuisse gauche sur dissection artérielle iatrogène de l´artère fémorale superficielle ipsilaterale ont constitués les pathologies associées. Il sied de noter que la tuberculose pulmonaire et la grossesse au troisième trimestre sont cités parmi tant d´autres facteurs de risque ou de mauvais pronostic à la COVID-19 [23].

**Figure 6:**
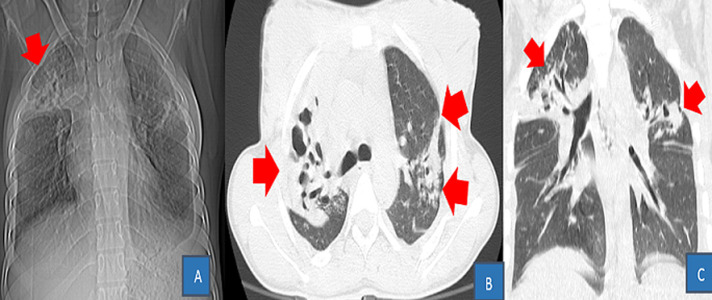
scanneur thoracique chez une gestante âgée de 19 ans avec coïnfection tuberculose pulmonaire, paludisme et COVID-19: A) vue coronale TDM en mode radio; B) vue axiale TDM en filtre parenchymateuse montrant une condensation bi lobaire supérieure; C) vue coronale TDM en filtre parenchymateux, montrant les foyers de condensation

Les formes cliniques observées chez les femmes enceintes ne diffèrent pas de celles observées chez les femmes non enceintes: la grossesse et la tuberculose étant des états de dépression immunitaire devant l´infection COVID-19 tempête de tous les types d´immunité (Figure 6). Des formes graves ont été rarement observées, avec la même fréquence que chez les femmes non enceintes. Les formes les plus symptomatiques, comme ceci est habituel dans les infections des voies aériennes supérieures aiguës, augmentent le risque d´accouchement prématuré, de retard de croissance ou d´accouchement par césarienne. Il faudra retenir que dans la présente étude, toutes les trois gestantes avaient bien évoluées, y compris celle porteuse de la coïnfection covid tuberculeuse et grossesse. Elles avaient toutes accouché à terme et les enfants étaient bien portants. Ce constat confirmerait d´avantage l´hypothèse selon laquelle le SARS-CoV-2 n´est pas présent dans les sécrétions vaginales ou dans le liquide amniotique. La dissection arterielle iatrogène de l´artère fémorale superficielle, peut être expliquée par la fragilité vasculaire qu´engendre l´infection à SARS-CoV-2. Cette fragilité vasculaire peut être due par la présence des taux élevés de cytokines circulantes ayant été rapportés chez les patients atteints de COVID-19 sévère entre autres (IL2, IL6, IL7, IL10, GSCF, IP10, MCP1, MIP1A et TNF) [24].

**Limitations de l´étude**: comme limitations; nous avons relevé une taille réduite de l´échantillon et un faible taux de patients ayant réalisé le CT scan thoracique sur un total de deux cents dix patients ayant été hospitalisé lors de la rédaction de ce papier. Les raisons sus évoquées ne permettent pas de généraliser ces résultats à l´ensemble de notre peuple.

## Conclusion

Les lésions TDM évocatrices de COVID-19, retrouvées chez patients hospitalisés aux CUK lors de la première vague étaient dominées par les opacités en verre dépoli. La majorité de nos patients avaient présenté précocement une forme sévère (étendue lésionnelle comprise entre: 50-75%). Nous pouvons enfin conclure que les aspects TDM retrouvés chez les patients hospitalisés aux CUK lors de la première vague de la pandémie à COVID-19; corroborent majoritairement les observations de la littérature. La propagation rapide du virus dans divers continents, l'augmentation du nombre de décès de jour en jour et la faible sensibilité de la PCR doivent conduire les praticiens à un diagnostic alternatif autre que le PCR; d´où l´intérêt de faire la TDM thoracique qui joue à ce jour un rôle majeur non seulement dans le triage rapide des patients dyspnéiques; mais surtout dans l´évaluation de l'extension lésionnelle, véritable facteur pronostic corrélé à la sévérité lésionnelle.

### Etat des connaissances sur le sujet

- La COVID-19 est causée par le coronavirus du syndrome respiratoire aigu sévère 2 (SARS-CoV-2). En décembre 2019, les premiers cas de COVID-19 ont émergé dans la région de Wuhan, en Chine, où des personnes ont manifesté des symptômes de pneumonie sévère;
- En janvier 2020, le virus s´est répandu à travers l´Asie, l´Europe, les Amériques et en le 11 mars 2020, l´OMS a déclaré l´état de pandémie alors que 114 pays dénombraient des cas de la maladie;
- La RT-PCR reste la biologie de référence quant à sa spécificité, tan disque le CT Scan thoracique reste la modalité d´imagerie la plus sensible avec comme lésions typiques de la maladie: des opacités en verre dépoli, des opacités en crazy paving ainsi que des condensations parenchymateuses non systématisées.

### Contribution de notre étude à la connaissance

- Cette étude; est une première en milieux hospitaliers de Kinshasa (CUK en particulier); à décrire les aspects TDM typiques et atypiques de SARS-CoV-2 chez les congolais
- Elle a prouvée l´existence des quelques spécificités tel que les épanchements pleuropéricardiques, les adénopathies des chaines parietales et viscerales, le nodule angiocentré sans oublié la miliaire tuberculeuse dans la coïnfection COVID-19-Tuberculose pulmonaire post primaire et paludisme chez une jeune gestante de 19 ans.
